# Preliminary study of the accuracy and safety of robot-assisted mandibular distraction osteogenesis with electromagnetic navigation in hemifacial microsomia using rabbit models

**DOI:** 10.1038/s41598-022-21893-y

**Published:** 2022-11-15

**Authors:** Ziwei Zhang, Byeong Seop Kim, Wenqing Han, Mengzhe Sun, Xiaojun Chen, Yingjie Yan, Haisong Xu, Gang Chai, Li Lin

**Affiliations:** Department of Plastic and Reconstructive Surgery, Shanghai Ninth People’s Hospital, Shanghai JiaoTong University School of Medicine, 639 Zhi Zao Ju Road, Shanghai, 200011 China

**Keywords:** Surgery, Preclinical research

## Abstract

This study aimed to investigate the accuracy and safety of mandibular osteotomy and distraction device positioning in distraction osteogenesis assisted by an electromagnetic navigation surgical robot. Twelve New Zealand white rabbits were randomly divided into two groups after computed tomography. The control group underwent a procedure based on the preoperative three-dimensional design and clinical experience. Animals in experimental group underwent a procedure with robotic assistance after registration. The accuracies of osteotomy and distraction device positioning were analysed based on distance and angular errors. The change in ramus length after a 1 cm-extension of the distraction device was for assessing distraction effect. The preparation, operative and osteotomy times, intraoperative bleeding, and teeth injury were used for safety assessment. In the experimental group, the distance (t = 2.591, p = 0.011) and angular (t = 4.276, p = 0.002) errors of osteotomy plane, and the errors in distraction device position (t = 3.222, p = 0.009) and direction (t = 4.697, p = 0.001) were lower; the distraction effect was better (t = 4.096, p = 0.002). There was no significant difference in the osteotomy time and bleeding; however, the overall operative and preparation times were increased in the experimental group, with a reduced rate of teeth damage. Robot-assisted mandibular distraction osteogenesis with electromagnetic navigation in craniofacial microsomia is feasible, safe, significantly improves surgical precision.

## Introduction

Hemifacial microsomia is one of the most common congenital craniofacial anomalies after cleft lip and palate, with an incidence of approximately 1/5600–1/3000^1^. Hemifacial microsomia is characterised by hypoplasia of the mandible as the primary clinical manifestation, often accompanied by deformities of the orbit, ear, facial nerve, and soft tissues^2^. Previous studies have reported the successful treatment of patients with mandibular distraction osteogenesis, which has become a reliable method for treating hemifacial microsomia^3,4^. As the preferred surgical treatment for hemifacial microsomia, distraction osteogenesis allows the previously underdeveloped mandibular ramus to grow new bone in the direction and length designed by the surgeon preoperatively, while the shape and final morphology of the mandible are determined by the position and direction of the distraction device^5,6^. Therefore, ensuring osteotomy precision and accurate distraction device placement is essential for achieving clinical success. Although its application is well established and widespread, it is highly dependent on the experience of the attending surgeon. Operations on patients with hemifacial microsomia may be performed when patients are young, with a complex surrounding anatomy, and in a narrow surgical operating space^7^. Insufficient operative precision leads to adverse consequences such as damage to the dental germ and neurovascular injury^8^ and deviations in the length and direction of postoperative mandibular ramus distraction due to inconsistency between the actual outcome and the surgical design, which ultimately affects the morphological symmetry and functional recovery of the mandible. The precise and safe implementation of mandibular distraction remains a clinical bottleneck in the surgical treatment of patients with hemifacial shortening.

Computer-assisted technology has been widely developed and applied in complex craniofacial reconstruction in recent decades, and the intraoperative use of navigation has been attempted to improve surgical precision and reduce surgical complications^9–11^. The most common type of optical navigation is susceptible to intraoperative optical obstruction^12^ and is therefore limited in situations with a narrow intraoral field or complex instrumentation; additionally, muscle or soft tissue interfere with osteotomy guide plate placement, commonly performed during craniomaxillofacial surgery, thus affecting surgical accuracy. These clinical issues require further research.

Surgical robotics can significantly reduce manual errors by the surgeon due to its stable and precise movements when combined with the emerging technology of electromagnetic navigation, which allows convenient and flexible tracking of surgical objects and instruments by sensing the position of coils in a magnetic field independent of light^13,14^. The application of electromagnetic navigation surgical robots is becoming established^15^. In craniomaxillofacial surgery, studies initially demonstrated that intelligent surgical robots using magnetic navigation can assist osteotomy of the mandible by aiding the intraoperative trajectory planning with an accuracy that meets clinical needs^16^. However, there have been no studies on the application of electromagnetic navigation surgical robots in mandibular ramus distraction osteogenesis for hemifacial microsomia.

Based on previous robot-assisted mandibular surgeries^16–19^, we conducted a preclinical feasibility study to investigate the surgical outcomes of electromagnetic navigation robot-assisted osteotomy and distraction device positioning and to assess the accuracy of the surgery by comparing postoperative computed tomography (CT) with preoperative CT designs.

## Results

The surgery and postoperative CT examinations were completed in both groups. The height of the right mandibular ramus was measured and recorded preoperatively and after 1 cm of lengthening of the distraction device. There was a significant difference between the two groups (t = 4.096, p = 0.002, Table 1).

**Table 1:** Postoperative results and accuracy assessment.

	Length change of mandibular ramus (mean ± SD, mm)	Osteotomy distance error (mean ± SD, mm)	Osteotomy plane angular error (mean ± SD, °)	Distraction device positional error (mean ± SD, mm)	Distraction device directional error (mean ± SD, °)
Exp. animals	8.62 ± 0.90	1.99 ± 1.32	9.73 ± 3.56	4.79 ± 7.81	6.51 ± 3.19
Ctrl. animals	6.57 ± 0.84	2.61 ± 1.30	18.97 ± 3.92	7.81 ± 1.58	15.07 ± 3.13
	t = 4.096, p = 0.002	t = 2.591, p = 0.011	t = 4.276, p = 0.002	t = 3.222, p = 0.009	t = 4.697, p = 0.001

*Exp.* Experimental, *Ctrl.* control.

### Analysis for accuracy of osteotomy

The difference between the preoperative design and the postoperative mandible was visualised in the 3D colour map generated after fitting in Geomagic Control. The results of the distance error of the osteotomy line and the angular error of the osteotomy plane are shown in Table 1. There was a significant difference in the distance error (t = 2.591, p = 0.011); the angular error of the osteotomy plane was also significantly smaller in the experimental group than in the control group (t = 4.276, p = 0.002). Although the accuracy in the experimental group was only improved by approximately 0.62 mm, numerically, the robot system reduced the distance error by 23.8% (1.99 mm versus 2.61 mm); on the other hand, the angular error of the osteotomy plane was reduced by 48.7% (9.73° versus 18.97°).

### Analysis for accuracy of distraction device fixation

The results of the distance error analysis of the distraction device positional and directional errors assessment are displayed in Table 1. The distraction device positional error (t = 3.222, p = 0.009) and directional error (t = 4.697, p = 0.001) in the experimental group were smaller than those in the control group. Correspondingly, the positional error of the robot-assisted distraction device positioning was reduced by 38.7% (4.79 mm versus 7.81 mm) and by 56.8% in the directional error (6.51° versus 15.07°), compared to the control group. Our analyses suggested that the electromagnetic navigation robot system was effective in increasing the accuracy of distraction osteogenesis.

### Analysis for safety and preparation

The data on the operative, osteotomy, and preparation times; bleeding; and teeth injury are summarised in Table 2. The preparation time for each animal was approximately 45 min, including the production and fixation of the registration complex, fixation of the animal's mandible, and registration time before the surgery. Due to the additional handling required compared to conventional surgery, the operative time was significantly longer in the experimental group (t = 6.649, p < 0.001), and the animals were anaesthetised for a correspondingly longer period. However, there was no significant difference in intraoperative bleeding (t = 0.415, p = 0.687) and osteotomy time (t = 0.405, p = 0.694) between the two groups. The animals in the experimental group showed no dental injury, while in the control group, there were two cases where the root of the molar was damaged by the osteotomy and the screws holding the distraction device in place.

**Table 2:** Safety and preparation assessment.

No. of the animals	Ctrl. animals				Exp. animals				
	Operative time (min)	Osteotomy time (min)	Intraoperative bleeding(ml)	Teeth injury	Operative time (min)	Osteotomy time (min)	Intraoperative bleeding(ml)	Teeth injury	Preparation time (min)
1	60	5	5	–	90	7	10	–	45
2	70	5	10	^†^	100	6	5	–	
3	75	8	10	–	120	8	15	–	
4	55	5	5	^‡^	100	5	10	–	
5	65	4	5	–	110	5	5	–	
6	60	6	10	–	90	4	5	–	
Mean ± SD	64.17 ± 7.36	5.50 ± 1.38	7.50 ± 2.74		101.67 ± 11.69	5.83 ± 1.47	8.33 ± 4.08		

^†^Osteotomy line cutting the molar.

^‡^One screw damaging the dental root during the distraction device fixation.

All animals survived for one week postoperatively, without serious adverse effects. This is a preliminary indication of the safety of robot-assisted surgery.

## Discussion

Robot-assisted systems have prospects for application in complex craniomaxillofacial surgery because of their precision and stability. This study used robotic-intelligent navigation to assist in the guidance of the osteotomy and distraction device placement in mandibular distraction osteogenesis. The results verified that electromagnetic navigation robot-assisted distraction osteogenesis for hemifacial microsomia has good precision and safety in animal experiments.

In this study, we compared electromagnetic navigation robot-assisted surgery with a conventional surgical approach. The results showed that the osteotomy accuracy of both the conventional and robot-assisted surgery met the clinical requirements, and the distance errors of the osteotomy in both groups (1.99 ± 1.32 mm in the experimental group and 2.61 ± 1.30 mm in the control group) were comparable to the thickness of the osteotomy saw blade. Moreover, in terms of the distraction direction, robot-assisted distraction device positioning could effectively reduce the error due to external manual factors and further reduce the error between the actual postoperative distraction direction and the ideal direction designed preoperatively compared to conventional surgery, thereby achieving a higher possible increase in the height of the ramus of the dysplastic mandible. This was also suggested by the results of the ramus height change in this study. Our results confirmed the accuracy of the electromagnetic navigation robot system and its effectiveness in reducing intraoperative manual errors. The errors in the results of this study might have arisen from the thickness of the CT scan layers, magnetic field instability during electromagnetic navigation or interference from metallic instruments, and slight intraoperative mandibular displacement.

The application of digital technology has dramatically boosted craniomaxillofacial surgery: computer-aided design, virtual surgical planning, rapid prototyping techniques, intraoperative navigation, and other techniques significantly improve surgical precision and reduce complications. Watzinger et al. first reported on CAD techniques in patients with mandibular dysplasia^20^. The planning of a unidirectional intraoral distraction device implantation yielded good postoperative results. In subsequent studies, investigators designed different guides using CAM technology and successfully translated the CAD protocol into clinical practice, enabling intraoperative navigation. Yu et al. demonstrated the feasibility and accuracy of dental splints to assist the osteotomy and distractor fixation in a small sample of consecutive patients^21^. Recently El Hadidi et al. conducted a randomized controlled trial to verify that CAD/CAM guides increase the accuracy of the procedure^22^. In applying of navigation systems, Cai et al. validated the accuracy of the TBNavis-CMFS navigation system in the goat mandibles with optical navigation^23^.

By reviewing the optical navigation systems with frame devices, we found the view might be obstructed, and the surgical instruments obstruct signal transmission. Therefore, simple electromagnetic navigation could be more suitable for a limited field of view, as in intraoral incisions with small surgical spaces. Additionally, the conventional navigational process requires the operator to pay attention to both the virtual interface display and the real surgical scene, which requires the operator to switch the view frequently^23^. The virtual data in this study could be transferred accurately in real space without the need for the operator to change focus, making the procedure safer.

There are also several problems with the widely used guide plate technique for distraction osteogenesis. The anatomical characteristics of the mandibular ramus do not allow for firm fixation of the plate, which is exacerbated by inevitable bleeding during the surgery; furthermore, the soft tissues attached to the bone make it difficult for the plate to fit the bone tightly, leading to a reduction in the accuracy of the osteotomy after installation^11^. In addition, an inadequacy in the design of the plate or the installation process may cause mounting difficulties or soft tissue damage^12^. The previous clinical study also found more dissection and retraction to fit the guide plates^22^. The robot-assisted technique could reach the precise position of the preoperative design, thereby overcoming the deficiencies of the plate.

To achieve precise and stable dual positioning for osteotomy and distraction device placement in MDO under high-stress surgical conditions, we used the robotic electromagnetic navigation system, which integrates multiple digital technologies such as CAD, 3D imaging processing, and intraoperative navigation, to achieve commanded movements in a restricted field of the deep intraoral incision.

Electromagnetic navigation has been successfully used in bronchoscopy and vascular interventions^24–26^, and its convenience and flexibility have been demonstrated. Previous studies had concluded that the accuracy of electromagnetic navigation in craniomaxillofacial surgery could meet the clinical needs and that the accuracy of assisted osteotomy in craniotomy and orthognathic surgery could equal that of optical navigation^27,28^. We have developed an effective tool for accurately translating virtual preoperative designs to the actual surgical environment by combining electromagnetic navigation with a surgical robot.

The application of electromagnetic navigation requires the collaboration of objects in reality with the virtual coordinate system of the surgical plan^13^. In this study, we used a minimally invasive approach to fix the marker to the bone, and no significant intraoperative bleeding or soft tissue trauma was observed. Compared to body surface markers for calibration^29^, skeletal fixation is less affected by the external forces of intraoperative manipulation and is more stable. Automatic registration via small steel balls on the registration complex not only simplifies the navigation process and reduces surgical exposure but also eliminates inaccuracies in the manual selection of registration points and increases the accuracy even further.

The surgical preparation for robot-assisted distraction osteogenesis, took approximately 45 min, and the surgery was longer than in the control group (Table 2). The additional time during the surgery included time spent for robotic registration and waiting for the robot end with the template to be in place; there was no significant difference in the osteotomy time between the two groups, and there was no increased risk of intraoperative bleeding in the experimental group.

Robot-assisted distraction osteogenesis with electromagnetic navigation improves surgical precision in this study, but it has limitations. First, more preparation is necessary before surgery. The production of the registration complex requires a lead time, and the use of the robot increases the operative cost, which increases the financial burden on the patient. Appropriate robot positioning during surgery also requires more space in the operating room, and there may be interference between devices during surgery. There are also limitations to the experimental design of this study. The sample size of the animal experiments was small, although the statistical results showed significant differences. We would still like to conduct experiments with larger sample sizes to further validate these findings. Additionally, this study used an extra-oral approach in animals to verify the accuracy and safety of robot-assisted surgery, which differs from the clinical intraoral incision approach. In the assessment of adverse events, we chose the objective presentation of dental damage, which was with limitation. Studying the function of the facial nerve or the movement of the expression muscles in animals is rather difficult because the animals are unable to follow the instructions from the researchers or produce expressions.

Therefore, although we performed the surgery successfully in animals and obtained satisfactory results, there are still issues requiring improvements before clinical applications. We will continue to work on further reducing environmental errors such as electromagnetic interference and registration errors within the robot system; optimizing the robot algorithm workflow to obtain a better solution for execution path planning; we will also aim for a more user-friendly software operation and a more flexible overall appearance. In terms of follow-up trial design, we should also consider that in clinical practice, patients with severe mandibular dysplasia can have functional deficits of the temporomandibular joint, and trials proposed to include this group of patients should include evaluations of the temporomandibular joint. In addition, analysis of longer follow-up results would facilitate the further assessment of postoperative outcomes.

## Conclusion

As the primary surgical treatment for hemifacial microsomia, the precise position and direction of the osteotomy and distraction need to be ensured, so the accurate implementation of the preoperative design is particularly critical. In this study, all surgeries were successfully performed on all animals using the electromagnetic navigation robot. A comparison of postoperative CT with the preoperative design CT initially demonstrated the feasibility and safety of electromagnetic navigation robot-assisted distraction osteogenesis for hemifacial microsomia. The assisted osteotomy and assisted distraction device placement accuracy were higher than that of the conventional surgical group. The surgical outcomes were also better in the experimental group. The results of this study suggest that this technology has clinical application prospects and can be applied to improve surgical precision and postoperative distraction results.

## Methods

### Study protocol

Twelve healthy male New Zealand white rabbits (weighing 2.5–3.5 kg) were randomly assigned to the experimental and control groups in a 1:1 ratio using a random number table. The sample size was calculated using Eq. (1).

1 $$n={\left[\frac{{Z}_{\frac{\alpha }{2}}+{Z}_{\beta }}{\delta /\sigma }\right]}^{2}({Q}_{1}^{-1}+{Q}_{2}^{-1})$$

**Figure 1:**
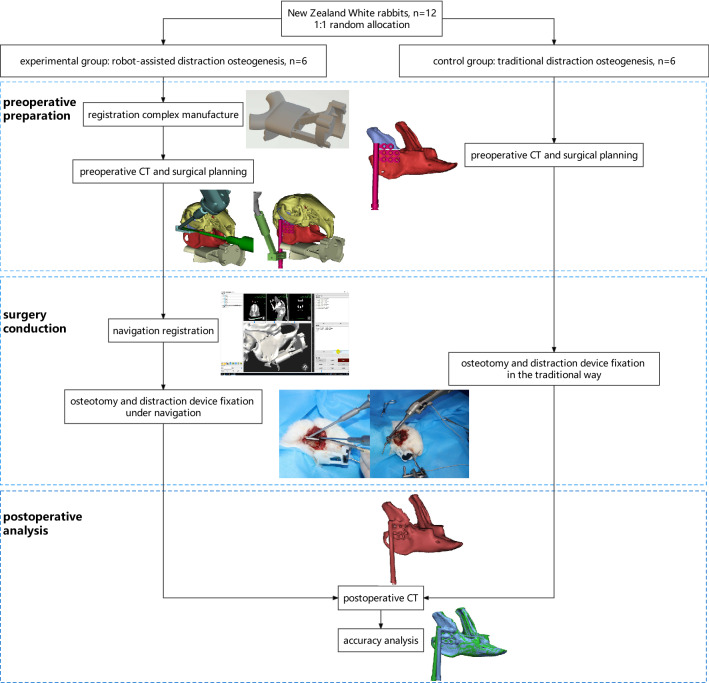
Overall workflow diagram.

In this study, n1 = n2, so Q1 = Q2 = 0.5. Power (1 − β) was set as 0.9 and α as 0.05. Empirically a difference of approximately 2 mm was derived and the standard deviation was taken as 1.03 mm based on the results of previous animal experiment of robot-assisted osteotomy using electromagnetic navigation (16). So n = ${\left[\frac{1.96+1.28}{2/1.03}\right]}^{2}({0.5}^{-1}+{0.5}^{-1})\approx 11.14$, and n1 = n2 should at least be 6. The evaluators were blinded, but the investigators were not, because the experimental and control groups underwent different procedures. The operating surgeons were all craniomaxillofacial specialists with at least five years of surgical experience. All animals were treated on the right side. The animals in the experimental group underwent distraction osteogenesis with the assistant of a craniomaxillofacial surgical navigation system, while the control group underwent mandibular ramus osteotomy and distraction device fixation using conventional surgical methods (carrying out the preoperative plan based on clinical experience) (see flowchart, Fig. 1). All experiments were performed in accordance with the revised Animals (Scientific Procedures) Act 1986 or comparable ethical standards. We conducted the study in accordance with the ARRIVE checklist. This study was approved by the Ethics Committee for experimental animals of the Shanghai Ninth People's Hospital, Shanghai Jiao Tong University School of Medicine (SH9H-2021-A969-SB).

### Preoperative preparation

#### Manufacturing the registration complex

**Figure 2:**
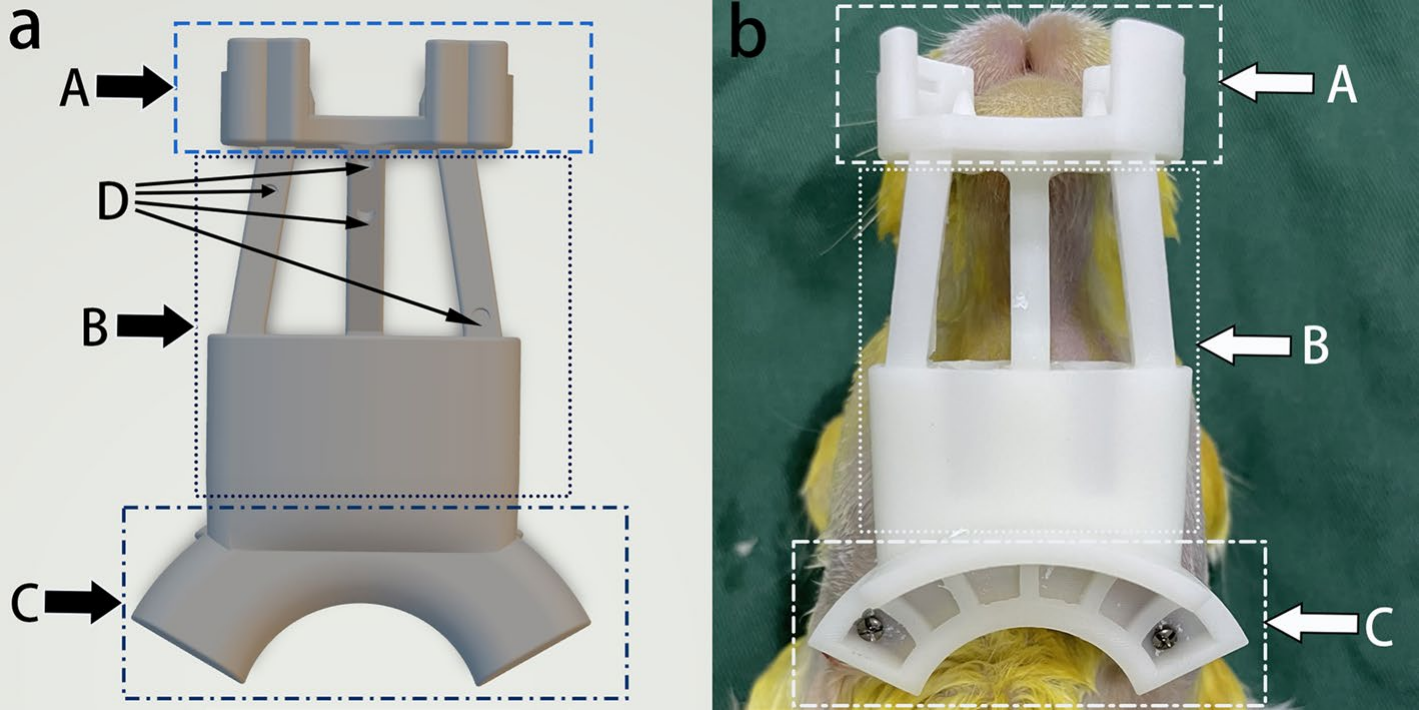
Registration complex. (**a**) The design schematic of the registration complex (view from the dorsal angle). (**b**) 3D-printed registration complex fixed on the rabbit mandible (view from the ventral angle). (A) fixation module, (B) connected module, (C) pedestal for the electromagnetic sensor, (D) positions to install steel balls.

In this study, we developed a registration complex consisting of three parts: a fixation module, a connected module, and a pedestal for the electromagnetic sensor (Fig. 2). The fixation module was pinned to the mandible of the animal, and the connected module had four 2 mm diameter steel balls fixed to it, which served as navigation markers for magnetic navigation registration. The electromagnetic sensor was then tightly fixed to the pedestal. Preoperative CT (Brilliance 64 CT scanner, Philips, the Netherlands) of the animal with the registration complex correctly fixed was performed.

#### Preoperative design

**Figure 3:**
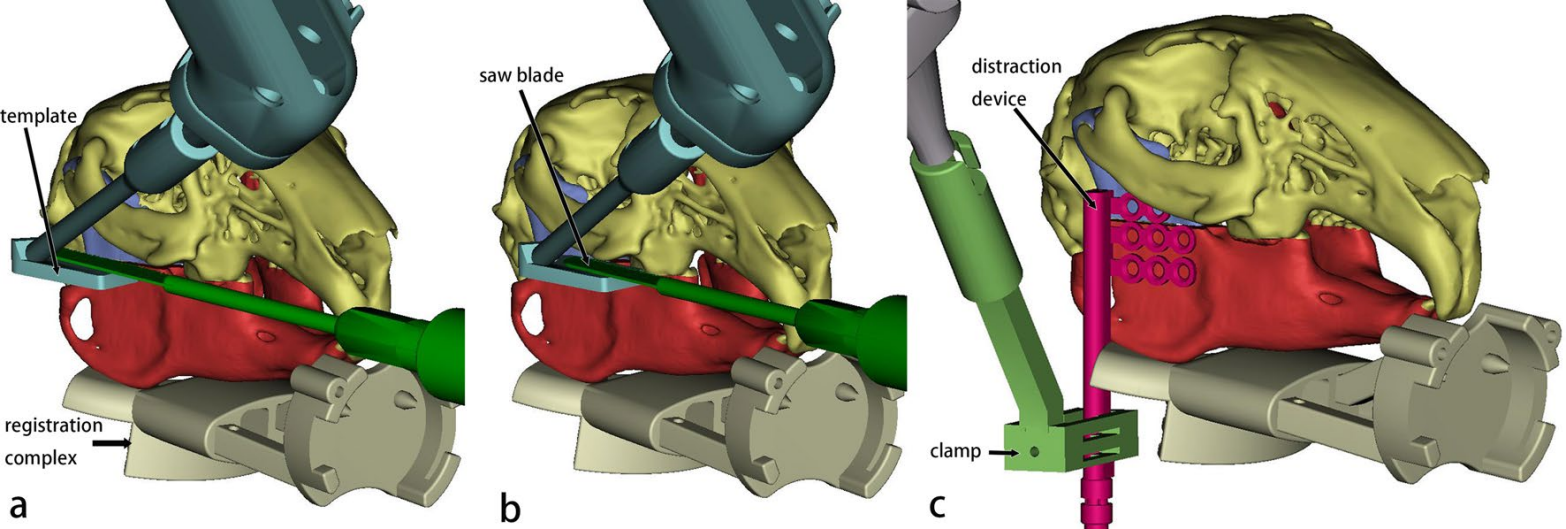
Surgical plan shown in Mimics. (**a**) The template is at the initial position to guide the osteotomy, with the saw blade closely beside it. (**b**) The template is at the second position, and the blade moves forward along it. (**c**) The clamp with the distractor on is in position. Cyan: robotic arm end with the template. Green: reciprocating bone saw. Light gray: registration complex. Light green: self-designed clamp. Pink: distractor. Purple: the proximal mandibular ramus of the osteotomy line. Red: the distal part of mandible.

The preoperative CT was saved in Digital Imaging and Communications in Medicine (DICOM) format and later imported into the 3D reconstruction software Mimics 21.0 (Materialise, Leuven, Belgium); the mandible was segmented and reconstructed. The osteotomy line and distraction device position were designed based on the vector of the mandibular ramus and the position of the molar root and inferior alveolar nerve, with the distraction device perpendicular to the occlusal plane^6,11^. Simultaneously, we considered the need to avoid obstacles during the navigation of the robot and to avoid interference with the use of surgical instruments, so we included a corresponding part for the simulation of osteotomies and the placement of distraction devices under robotic guidance in the preoperative design (Fig. 3). The reconstructed model of the mandible with the completed osteotomy design was exported in STL format and subsequently imported into the robotic system, allowing the relative position between the mandible and the registration complex to be recorded by the robot.

#### Preparation of animals and instruments

The registration complexes, robot-assisted surgical templates, and distraction devices were sterilised. The animals were fasted for 12 h before surgery and anaesthetised by intramuscular injection of xylazine hydrochloride (20 mg/kg). The surgical site was prepared and disinfected.

### Performance of the robot-assisted surgery

#### Robotic registration

**Figure 4:**
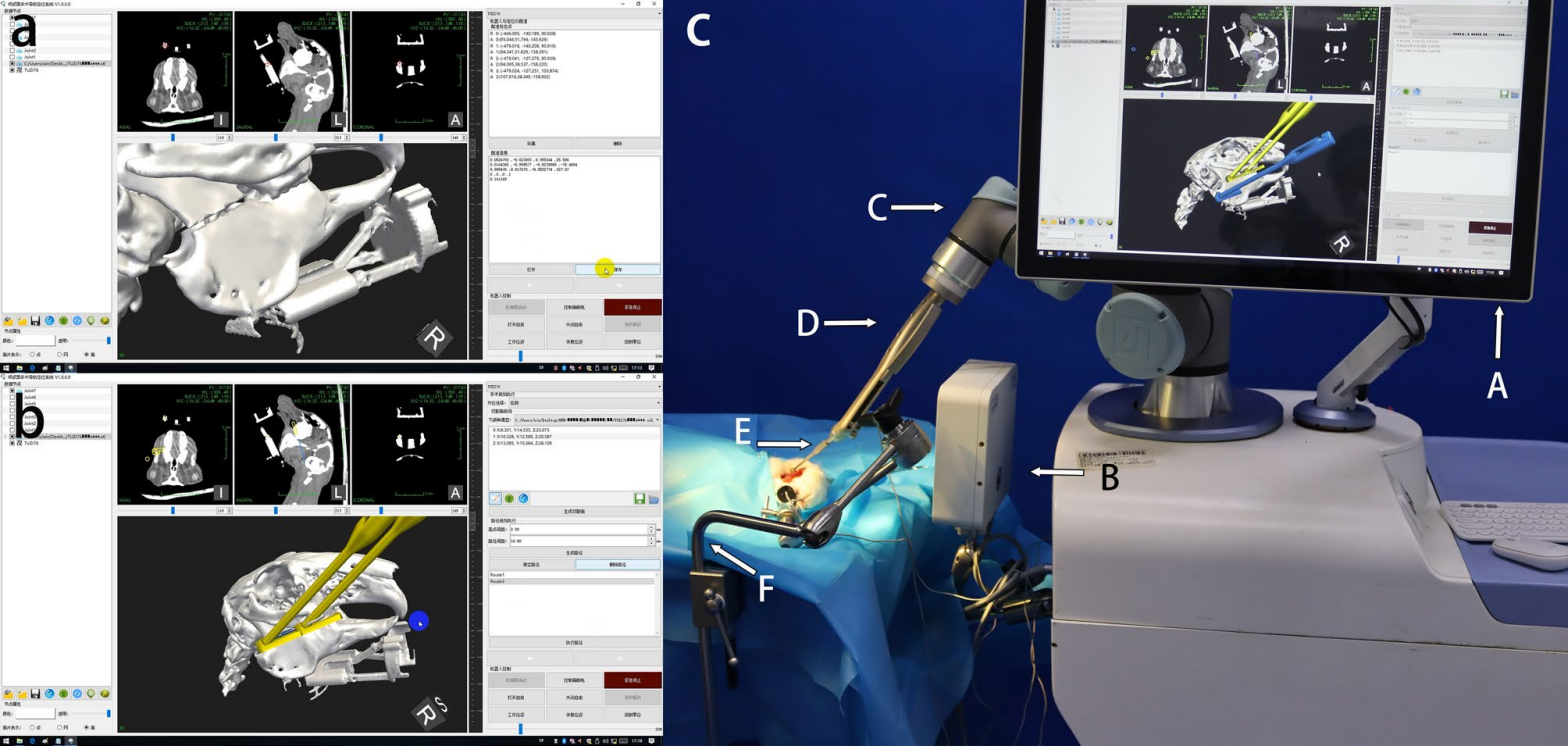
Robotic registration and initial positioning of the template. (**a**) Identification of the steel balls for registration, shown in the software. (**b**) The system calculates the movement path during osteotomy guidance (indicated in yellow), according to the preoperative design. (**c**) Panoramic view of the operating theatre. The robotic arm is ready to reach the target position automatically. The actual position of the template corresponds to the blue part. (A) computer screen, (B) electromagnetic generator, (C) robotic arm, (D) robotic arm sub-end for connection, (E) template, (F) fixator.

The instruments, including the robot electromagnetic navigation system, were positioned and switched on, and the surgical navigation software was turned on. The mandible of the experimental animals was fixed using a fixator (OZ 1251900, Berchtold, Germany). The designed template was connected to the robot arm. Magnetic field sensors were attached to the pedestal and end of the robot so that information on the relative position of the robot and mandible could be determined and tracked real time in the same magnetic field. Identifying the small steel balls on the registration complex in the electromagnetic field allowed the virtual data to collaborate with the actual position information via the matrix transformation. After registration, the system automatically calculated the path of movement of the template for the osteotomy guide based on the imported preoperative design data. The registration process and surgical path plan were visualised in the software as 3D images (Fig. 4a,b).

#### Robot-assisted osteotomy

The procedure was performed using an extra-oral approach with blunt dissection using a stripper to fully expose the mandibular ramus. Considering that the initial position of the robotic arm was far away from the target position, along the trajectory automatically calculated by the system, the robotic arm might hook onto nearby tissues during its movement causing unnecessary injuries or emergency braking due to accidental collisions.

**Figure 5:**
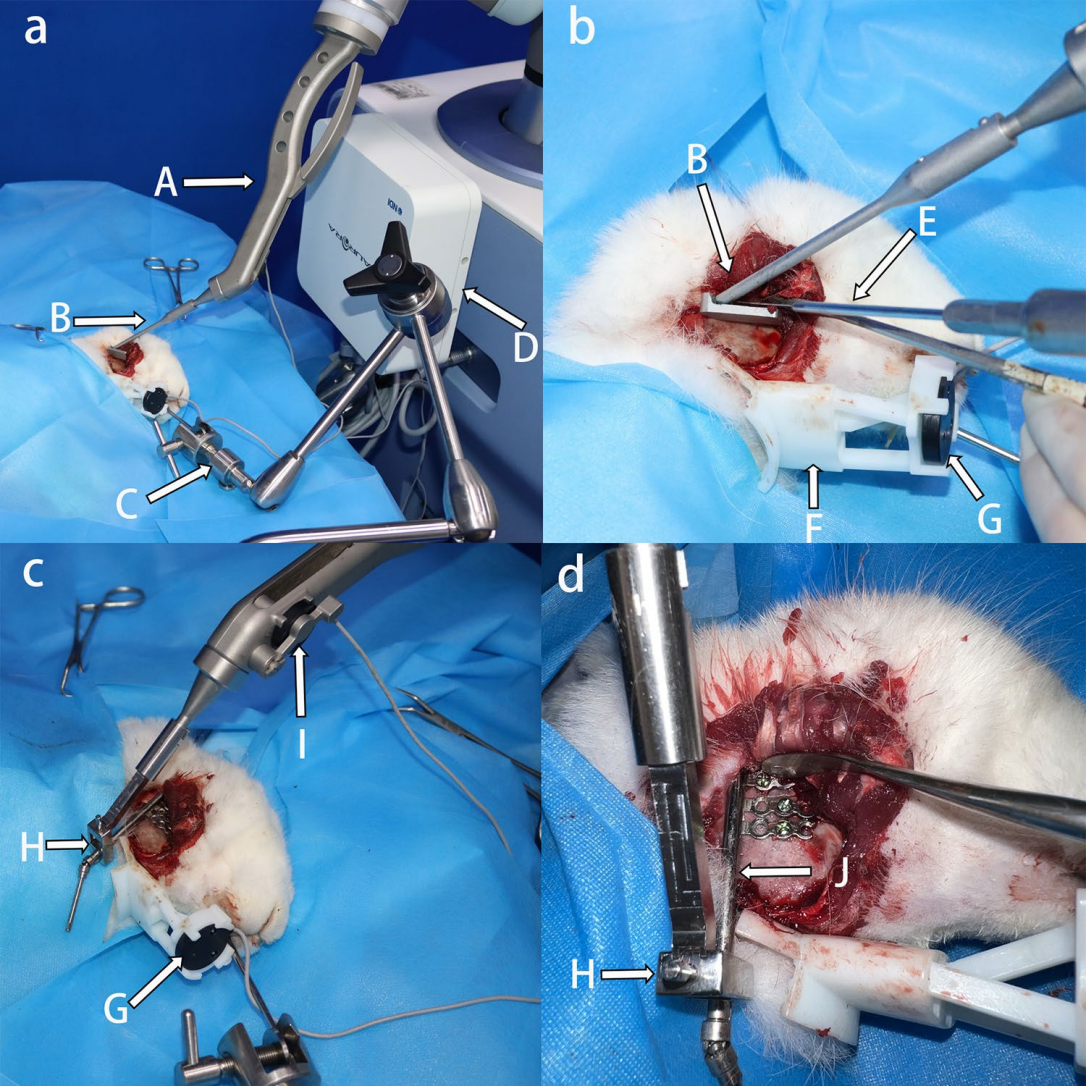
Robotic-assisted osteotomy and distractor positioning. (**a**) The template is at the initial target position. (**b**) Robotic-assisted osteotomy under electromagnetic navigation. The saw blade is against the template. The robot moves continuously from the initial position to the second position along the osteotomy line, with the saw blade following it forward. (**c**) The template has been replaced with the clamp, which holds the distractor in motion to the expected position. (**d**) Distractor implantation is completed after drilling and tightening the screws. (A) robotic arm sub-end, (B) template, (C) fixator, (D) electromagnetic generator, (E) bone saw, (F) registration complex, (G) electromagnetic sensor connected to the mandible, (H) clamp, (I) electromagnetic sensor connected to the robotic arm, (J) distractor.

Therefore, before the robot carried out automatic navigation, the operator moved the robotic end to the nearest position under the free mode (Fig. 4c), The free mode was then switched off, and the robot was ordered to follow the path. The template first reached the initial position (Fig. 5a). Holding the bone saw, the surgeon began the osteotomy close to the template (Fig. 5b).

Then the robotic template moved along the designed path at a constant speed, until the template moved to the second position and ended its movement. The saw blade moved forward with the template, and the osteotomy was finished with a complete osteotomy line.

#### Robot-assisted distraction device placement

After the osteotomy, the free mode was switched on again. The surgeon lifted the robotic arm slightly off the mandibular surface, removed the template, and replaced it with the distraction device to the robotic arm with a special clamp. The surgeons switched the object of operation in the software, after which the system calculated the path of the distraction device positioning process, which the operator commanded the robot to execute. The robot could be precisely positioned by magnetic navigation, and the arm was fixed in position based on the preoperative design (Fig. 5c).

Holding the drill, the surgeon drilled holes into the distraction device. The device was stabilised on the ramus using titanium nails (Fig. 5d). From the start of the registration to the end of the procedure, the positions of the robot base and the animal's mandible were fixed. The end of the robot was stationary after positioning and was not affected by external forces during drilling.

After the distraction device was placed, the clamp was removed, and the robotic arm was lifted under the free mode and then retracted automatically. For the animal, haemostasis was achieved, and the incision at the lower jaw was tightly sutured. Finally, the wound was cleaned and bandaged.

### Postoperative care for animals

The animals were kept in a suitable environment with adequate nutrition for one week after surgery. All the animals received a semi-liquid diet, intramuscular penicillin (500,000 U/kg, bid for 7 days) to combat infection, and subcutaneous buprenorphine (0.02 mg/kg, bid for 2 days) for analgesia.

### Postoperative analysis

All animals underwent postoperative 3D-CT under anaesthesia. The first image was taken immediately after distraction implantation, and the second was taken after 1 cm of distraction in situ. DICOM data were imported into Mimics, the mandible was reconstructed, and an STL file was saved. The postoperative image and preoperative design data were imported into Geomagic Control software (3D Systems, USA) for automated alignment by a researcher unaware of the test group.

**Figure 6:**
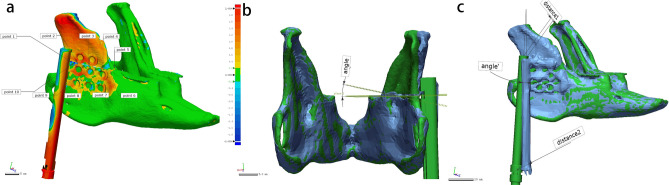
Accuracy analysis of osteotomy and distractor implantation. (**a**) Evaluation of the positional error according to ten points on the osteotomy plane. (**b**) Evaluation of the angular error according to the osteotomy planes. (**c**) Evaluation of the positional error according to the endpoints of the distractor’s driver screw, and evaluation of the distraction direction deviation using the lines through the endpoints.

The osteotomy accuracy of the two groups was evaluated based on the distance and angular errors of the mandibular osteotomy plane. The value of the distance error was obtained by calculating the average of the distance errors at 10 points (randomly selected) on the osteotomy plane between the postoperative image and preoperative design (Fig. 6a). The angular error was obtained by calculating the angle between the two osteotomy planes (Fig. 6b). The accuracy of the distraction device fixation was assessed by the positional and directional errors of the distraction device. The positional error was obtained by calculating the average of the differences in the distance of the two endpoints of the device’s driver screw.

The directional error was analysed by measuring the angular difference between the postoperative data and preoperative design simulation of the distraction directions (the direction of the line through the two endpoints) (Fig. 6c). Postoperative results after distraction were assessed by the change in length of the mandibular ramus after a 1 cm extension of the distraction device, which was defined as the distance between the most superior point of the condyle and the gonion^6^ (the most inferior, posterior, and lateral points on the external angle of the mandible). It was measured in Mimics based on 3D-reconstructed images. All measurements were repeated three times for each case, and the mean value was taken as the final result for inclusion in the analysis.

To assess the safety of the surgery, the animals were closely monitored for postoperative complications. Additionally, we recorded the operative time, osteotomy time, and intraoperative bleeding. The operative time was defined as the time between the start of anaesthesia and the completion of all surgical procedures on the animals. The osteotomy time was defined as the time between the beginning and end of the osteotomy. Intraoperative bleeding was roughly estimated by the change in the weight of the gauze. Teeth injury was determined by observing whether the osteotomy or screw fixation has damaged the dental structure.

The experimental group required additional preparation, such as the production of the registration complex and robotic registration. The time required for all preparation procedures was also recorded.

### Statistical analysis

SPSS 26.0 (IBM Corp., Armonk, NY, USA) was used to perform the statistical analysis. A chi-square test was used to compare the complication rates between the groups. The Shapiro–Wilk test was used for normality and the Levene test for homogeneity of variance of the mandibular ramus length change, as well as osteotomy and distraction device placement errors, for both groups. If the normal distribution was satisfied and the variances were equal, the independent samples t-test was used, otherwise nonparametric tests was used to compare the differences. A statistically significant difference was set at α = 0.05 and p < 0.05.

## Data Availability

Correspondence and requests for materials and data should be addressed to G.C.
